# Echinacoside Ameliorates UVB-Induced Skin Damage Through Selective Inhibition of the Cutaneous TRPV3 Channel

**DOI:** 10.3390/molecules30092026

**Published:** 2025-05-02

**Authors:** Shilun Mo, Xinying Yue, Yaxuan Qu, Guoji Zhang, Liqin Wang, Xiaoying Sun

**Affiliations:** 1 Department of Natural Medicinal Chemistry and Pharmacognosy, School of Pharmacy, Qingdao Medical College of Qingdao University, 1 Ningde Road, Qingdao 266073, China; msl72862@163.com (S.M.); yxy198614@163.com (X.Y.); w13356359354@163.com (L.W.); 2 Department of Pharmacology, School of Pharmacy, Qingdao Medical College of Qingdao University, 1 Ningde Road, Qingdao 266073, China; 2020010071@qdu.edu.cn (Y.Q.); zhangguojiqdu@163.com (G.Z.); 3 Institute of Innovative Drugs, Qingdao University, 38 Dengzhou Road, Qingdao 266021, China

**Keywords:** acute skin injury, echinacoside, TRPV3, UVB

## Abstract

Excessive exposure to ultraviolet B (UVB) radiation can lead to skin damage, such as erythema and swelling. Echinacoside is a key effective ingredient of medicinal plant *Cistanche deserticola* commonly used for therapies and treatments for anti-aging and irradiation-related skin diseases. However, the molecular mechanism underlying the action of echinacoside remains unclear. Here, we report that echinacoside ameliorates UVB-induced skin damage by directly acting on the Ca^2+^-permeable and thermosensitive transient receptor potential vanilloid 3 (TRPV3) channel. Topical application of echinacoside efficaciously suppresses skin lesions induced by UVB radiation in wild-type mice but has no additional benefit in *Trpv3* knockout mice. In whole-cell patch clamp recordings, echinacoside selectively inhibits TRPV3 channel currents induced by 2-aminoethoxydiphenyl borate in a concentration-dependent manner with an IC_50_ value of 21.94 ± 1.28 μM. The single-channel patch clamp results show that echinacoside significantly reduces the open probability and open frequency without significantly altering TRPV3 channel unitary conductance. Molecular docking and site-specific mutagenesis indicate that residue T636 on the p-loop and residue T665 on the S6 segment of TRPV3 are critical for echinacoside binding to TRPV3. Taken together, our findings provide a molecular basis for further studies as use of natural echinacoside in irradiation-related skin care therapy, thus establishing a significant role of the TRPV3 channel in acute skin injury.

## 1. Introduction

The skin is a multifaceted organ composed of different cell types and structures, acting as the body’s primary barrier against external harm [1,2]. Ultraviolet radiation (UVR) is a common factor leading to skin injury [3,4]. At the same radiation exposure levels, ultraviolet B (UVB) exposure inflicts more significant harm to the skin compared to ultraviolet A (UVA) exposure. UVB is the main contributor to skin injury [5,6]. High-dose UVB exposure over a short period can lead to acute skin injury symptoms, including erythema, swelling, and pain [7,8]. Thus, preventing and treating UVB-induced skin damage is critically important.

In recent years, various bioactive compounds derived from medicinal plants have been used to treat UVB-induced skin damage [9,10,11]. *Cistanche deserticola* Y.C. Ma mainly grows in arid regions and warm deserts, and serves as the primary source of the traditional Chinese medicinal herb Cistanche [12]. In traditional Chinese medicine, it is commonly used to treat disorders related to the reproductive system and aging [13,14]. *C. deserticola* is praised for its excellent medicinal properties and is known as “Ginseng of the deserts” [15,16]. Echinacoside is the major constituent of the plant *C. deserticola* and serves as its crucial active ingredient [14,17]. Echinacoside has been shown to mitigate UVB-induced skin injury by eliminating reactive oxygen species and decreasing DNA injury [18]. Recently, echinacoside has also been shown to alleviate UVB-induced dermal fibroblast photoaging by activating the insulin-like growth factor-I signaling pathway [19]. However, the therapeutic mechanisms of echinacoside against UVB-induced skin injury have not yet been completely elucidated.

The transient receptor potential vanilloid 3 (TRPV3) channel is a Ca^2+^-permeable and thermosensitive nonselective cation channel abundantly expressed in skin keratinocytes [20,21,22,23]. TRPV3 has been widely demonstrated to play a crucial role in epidermal barrier repair and in various inflammatory skin diseases such as Olmsted syndrome, atopic dermatitis, and pruritus [21,24,25,26,27]. Recently, we have demonstrated that the TRPV3 channel plays a significant role in UVB radiation-induced acute skin lesions. Selective inhibition of TRPV3 activity may present a novel strategy for UVB radiation-induced skin damage treatment [28]. Our previous studies show that two phenylethanoid glycosides forsythoside B and verbascoside that can selectively inhibit the TRPV3 channel [29,30]. It is of interest that echinacoside shares a similar phenylethanoid core with both forsythoside B and verbascoside. Therefore, we hypothesized that echinacoside may ameliorate UVB-induced skin damage by directly acting on the TRPV3 channel.

In this study, we aim to investigate whether echinacoside alleviates UVB-induced acute skin injury through its action on TRPV3 and to elucidate the molecular determinants for the interaction between echinacoside and TRPV3. Our findings not only provide novel insights for echinacoside from the *C*. *deserticola* plant in irradiation-related skin care therapy, but also further confirm the critical role of TRPV3 in UVB-induced acute skin injury.

## 2. Results

### 2.1. Echinacoside Dose-Dependently Alleviates UVB-Induced Acute Skin Injury

**Figure 1:**
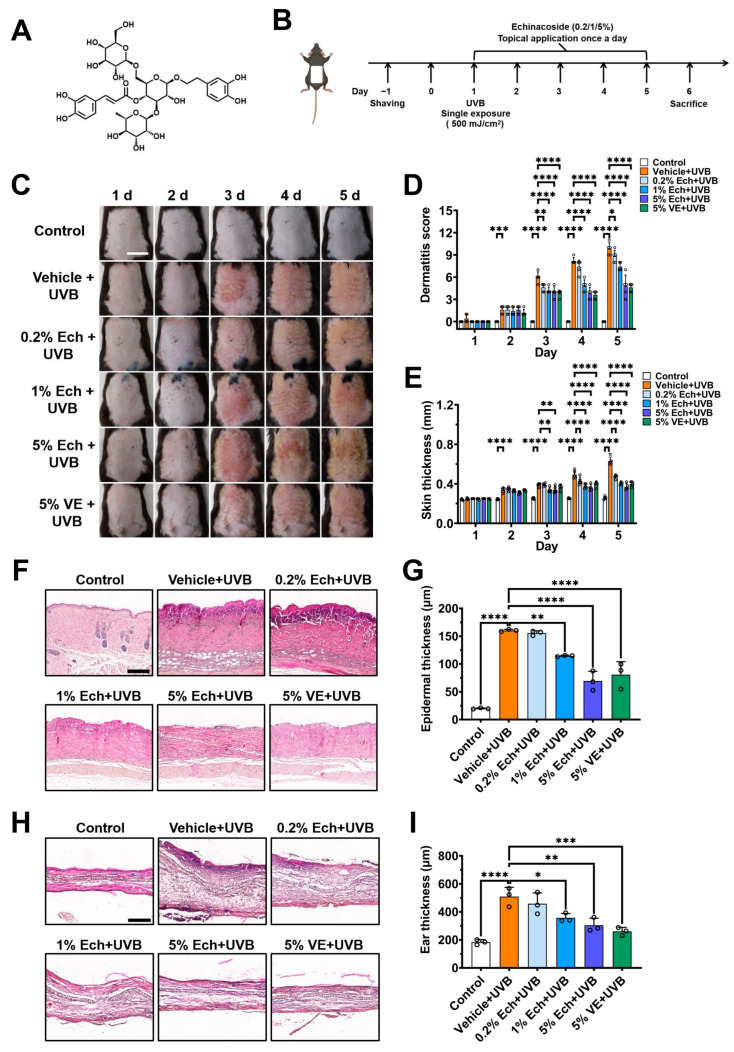
Concentration-dependent alleviation of UVB-induced dorsal skin and ear inflammation by echinacoside. (**A**) The chemical structure of echinacoside. (**B**) Schematic drawing of experimental procedures for generation of the mouse model of skin lesions induced by UVB radiation and treatment with echinacoside creams in different concentrations. (**C**) Representative images show the appearance of C57BL/6J mice dorsal skin after a single exposure to 500 mJ/cm^2^ UVB radiation and subsequent topical application of vehicle or different concentrations of echinacoside (Ech, 0.2, 1, 5%) or vitamin E (VE, 5%) creams for five consecutive days. Scale = 1 cm. (**D**) Quantitative analysis of dermatitis scores of the dorsal skin from panel (**C**) (*n* = 5, * *p* < 0.05, ** *p* < 0.01, *** *p* < 0.001, **** *p* < 0.0001, by two-way ANOVA followed by Dunnett’s multiple comparison tests). (**E**) Quantitative analysis of dorsal skin thickness from panel (**C**) (*n* = 5, ** *p* < 0.01, **** *p* < 0.0001, by two-way ANOVA followed by Dunnett’s multiple comparison tests). (**F**) Representative histology of paraffin-embedded mouse dorsal skin tissue sections (5 μm thickness) in hematoxylin and eosin (H&E) staining with infiltration of inflammatory cells and epidermal thickening on day 6. Scale = 200 μm. (**G**) Quantitative analysis of epidermal thickness from panel (**F**) (*n* = 3, ** *p* < 0.01, **** *p* < 0.0001, by one-way ANOVA followed by Dunnett’s multiple comparison tests). (**H**) Representative histology of optimal cutting temperature compound-embedded mouse ear tissue sections (8 μm thickness) in H&E staining with infiltration of inflammatory cells and thickening on day 6. Scale = 200 μm. (**I**) Quantitative analysis of ear thickness from panel (**H**) (*n* = 3, * *p* < 0.05, ** *p* < 0.01, *** *p* < 0.001, **** *p* < 0.0001, by one-way ANOVA followed by Dunnett’s multiple comparison tests). Data are expressed as the mean ± SD.

To examine the protective effect of echinacoside (Figure 1A) on acute skin injury induced by UVB radiation, we established the acute skin injury mouse model, in which C57BL/6J mice were exposed to a single dose of 500 mJ/cm^2^ UVB radiation, followed by immediate topical application of echinacoside creams at various concentrations (0.2, 1, 5%) to the mouse dorsal skin and ear once a day for five consecutive days (Figure 1B). A 5% vitamin E cream was used to serve as positive control. Phenotypic observations showed that topical application of echinacoside after UVB irradiation significantly alleviated UVB-induced mouse dorsal skin erythema and hyperplasia (Figure 1C). Additionally, the dermatitis scores of the dorsal skin were significantly reduced in both a concentration- and time-dependent manner (Figure 1D). On day 5, the dermatitis scores of mice dorsal skin treated with 0.2%, 1%, and 5% echinacoside decreased by 10.20% (*p* = 0.0337), 24.49% (*p* < 0.0001), and 51.02% (*p* < 0.0001), respectively, as compared with the vehicle group. In vernier caliper readings, echinacoside also reduced the dorsal skin thickness in a dose-dependent manner (Figure 1E). On day 5, the dorsal skin thickness of mice treated with 0.2%, 1%, and 5% echinacoside decreased by 25.40% (*p* < 0.0001), 36.51% (*p* < 0.0001), and 41.27% (*p* < 0.0001), respectively, as compared with the vehicle group.

Histological examination of dorsal skin and ear tissue sections on day 6 further confirmed that topical echinacoside at 0.2 to 5% alleviated UVB-induced the epidermal thickening, ear swelling, and skin inflammation in a concentration-dependent manner (Figure 1F–I). Compared with the vehicle group, the epidermal thickness of mice dorsal skin treated with 0.2%, 1%, and 5% echinacoside decreased by 2.89% (*p* = 0.9848), 28.56% (*p* = 0.0023), and 56.67% (*p* < 0.0001), respectively; the ear thickness of mice treated with 0.2%, 1%, and 5% echinacoside decreased by 10.01% (*p* = 0.6167), 29.86% (*p* = 0.0118), and 40.20% (*p* = 0.0013), respectively. These results indicate that echinacoside dose-dependently alleviates acute skin injury induced by UVB radiation.

### 2.2. Attenuation of UVB-Induced Acute Skin Injury by Echinacoside Is Dependent on TRPV3

**Figure 2:**
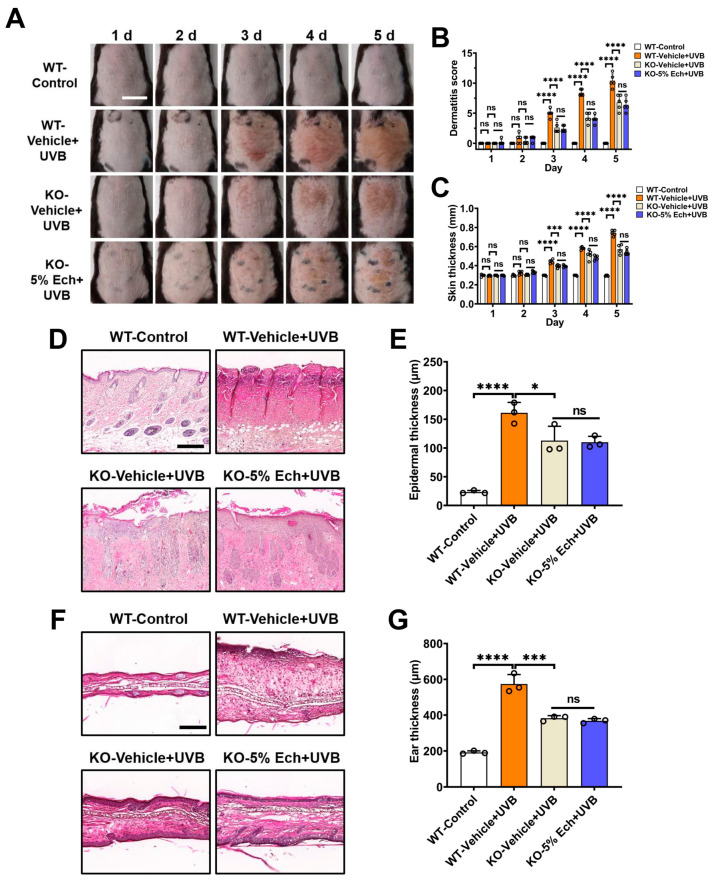
Attenuation of UVB-induced dorsal skin and ear inflammation by echinacoside is dependent on TRPV3. (**A**) Representative images show the appearance of dorsal skin in the wild-type (WT) and *Trpv3* knockout (KO) mice after a single exposure to 500 mJ/cm^2^ UVB radiation and subsequent topical application of vehicle or echinacoside (Ech, 5%) creams for five consecutive days. Scale = 1 cm. (**B**) Quantitative analysis of dermatitis scores of the dorsal skin from panel (**A**) (*n* = 5, ns, no significance, **** *p* < 0.0001, by two-way ANOVA followed by Dunnett’s multiple comparison tests). (**C**) Quantitative analysis of dorsal skin thickness from panel (**A**) (*n* = 5, ns, no significance, *** *p* < 0.001, **** *p* < 0.0001, by two-way ANOVA followed by Dunnett’s multiple comparison tests). (**D**) Representative histology of paraffin-embedded mouse dorsal skin tissue sections (5 μm thickness) in H&E staining with infiltration of inflammatory cells and epidermal thickening on day 6. Scale = 200 μm. (**E**) Quantitative analysis of epidermal thickness from panel (**D**) (*n* = 3, ns, no significance, * *p* < 0.05, **** *p* < 0.0001, by one-way ANOVA followed by Dunnett’s multiple comparison tests). (**F**) Representative histology of optimal cutting temperature compound-embedded mouse ear tissue sections (8 μm thickness) in H&E staining with infiltration of inflammatory cells and thickening on day 6. Scale = 200 μm. (**G**) Quantitative analysis of ear thickness from panel (**F**) (*n* = 3, ns, no significance, *** *p* < 0.001, **** *p* < 0.0001, by one-way ANOVA followed by Dunnett’s multiple comparison tests). Data are expressed as the mean ± SD.

To ascertain if the echinacoside-mediated effect is dependent on TRPV3, we utilized *Trpv3* knockout (KO) mice to assess the effect of TRPV3 silencing on UVB-induced acute skin injury. As shown in Figure 2A–C, knockout of the *Trpv3* gene attenuated UVB-induced mouse dorsal skin erythema, dermatitis score, and thickness, as compared with the wild-type (WT) vehicle group. The administration of 5% echinacoside cream did not have an additional effect on UVB-induced mouse dorsal skin erythema, dermatitis score (*p* = 0.6589), and thickness (*p* = 0.0573) in *Trpv3* KO mice, as compared with the *Trpv3* KO vehicle group on day 5. In addition, deletion of *Trpv3* also suppressed the UVB-induced epidermal thickening (*p* = 0.0200) and ear swelling (*p* = 0.0001) as compared with the WT vehicle group, and the application of 5% echinacoside cream had no additional protective effect on UVB-induced the epidermal thickening (*p* = 0.8681) and ear swelling (*p* = 0.2565) in the *Trpv3* KO mice (Figure 2D–G). These results indicate that silencing TRPV3 can prevent skin injury caused by UVB radiation, and the attenuation of UVB-induced acute skin injury by echinacoside is likely to act on TRPV3.

### 2.3. Echinacoside Is a Selective TRPV3 Inhibitor

**Figure 3:**
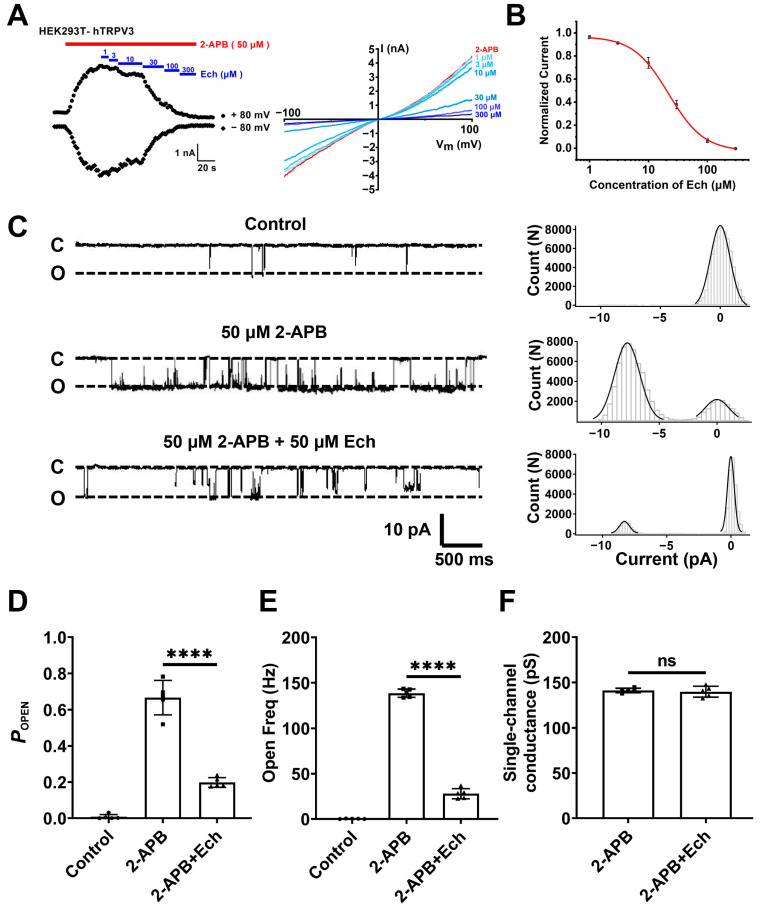
Echinacoside inhibits macroscopic and single-channel TRPV3 currents. (**A**) Left panel, the inhibitory effect of different concentrations of echinacoside (Ech) from 1 to 300 µM on human TRPV3 (hTRPV3) channel currents activated by 50 μM 2-APB. Right panel, current–voltage curves of hTRPV3 in response to voltage ramps from −100 to +100 mV from the left panel after the addition of 50 µM 2-APB and co-addition of echinacoside from 1 to 300 µM. (**B**) The concentration-dependent inhibition of hTRPV3 by echinacoside at +80 mV was analyzed by Hill equation fitting, with an IC_50_ value of 21.94 ± 1.28 µM (*n* = 5). (**C**) Left panels, representative single-channel current traces recorded at −60 mV in inside-out patch clamp recordings before and after the addition of 50 µM 2-APB or co-application with 50 µM echinacoside. All-point amplitude histograms of single-channel currents in 5 s are shown in their right panels. Dotted lines indicate the closed channel state (C) and the opened channel state (O), respectively. (**D**) Summary of the average open probability (*P*_OPEN_) values of hTRPV3 single channel in the presence of control (circle), TRPV3 agonist 2-APB (square), and co-application with echinacoside (triangles) (*n* = 5, **** *p* < 0.0001, by unpaired *t* test). (**E**) Summary of hTRPV3 single-channel open frequency (Freq) after exposure to control (circle), TRPV3 agonist 2-APB (square), and co-application with echinacoside (triangles) (*n* = 5, **** *p* < 0.0001, by unpaired *t* test). (**F**) Summary of hTRPV3 single-channel conductance after exposure to control (circle), TRPV3 agonist 2-APB (square), and co-application with echinacoside (triangles) (*n* = 5, ns, no significance, by unpaired *t* test). Data are expressed as the mean ± SD.

Based on the findings that echinacoside alleviates UVB-induced acute skin damage dependent on TRPV3, we examined its effect on TRPV3 currents using whole-cell patch clamp recording assay. In human embryonic kidney (HEK) 293T cells expressing human TRPV3 channels, echinacoside inhibited macroscopic TRPV3 currents induced by agonist 2-aminoethoxydiphenyl borate (2-APB, 50 μM) in a dose-dependent manner with an IC_50_ value of 21.94 ± 1.28 μM (Figure 3A,B). To further confirm the direct inhibitory effect of echinacoside on the TRPV3 channel, we recorded the single-channel currents in an inside-out configuration. When the agonist 2-APB (50 µM) activates the TRPV3 channel, it transforms the channel from the closed state (C) to the open state (O). After activation, the single-channel open probability (*P*_OPEN_) of TRPV3 increased to 0.67 ± 0.09, the open frequency (Freq) increased to 138.72 ± 4.44 Hz, respectively, and the single-channel conductance was 141.3 ± 2.4 pS. In contrast, echinacoside at 50 µM significantly reduced single-channel *P*_OPEN_ to 0.20 ± 0.03 (*p* < 0.0001) and open Freq to 27.89 ± 5.65 Hz (*p* < 0.0001), respectively, without any significant alteration of channel conductance of 139.92 ± 5.9 pS (*p* = 0.6440) (Figure 3C–F). These results indicate that echinacoside inhibits macroscopic TRPV3 currents by reducing the channel open probability and open frequency.

**Figure 4:**
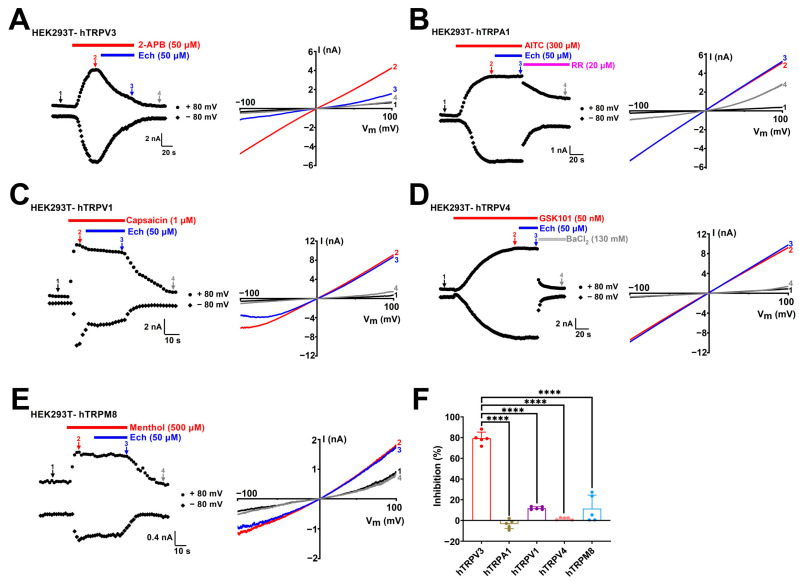
Selective inhibition of TRPV3 currents by echinacoside over other subtypes of thermo-TRP channels. (**A**–**E**) Left panel, whole-cell currents of human TRPV3 (hTRPV3) in response to 50 μM 2-APB (red bar) and co-application of 50 μM echinacoside (Ech, blue bar), human TRPA1 (hTRPA1) in response to 300 μM allyl isothiocyanate (AITC, red bar) and co-application of 50 μM echinacoside (Ech, blue bar), human TRPV1 (hTRPV1) in response to 1 μM capsaicin (red bar) and co-application of 50 μM echinacoside (Ech, blue bar), human TRPV4 (hTRPV4) in response to 50 nM GSK1016790A (GSK101, red bar) and co-application of 50 μM echinacoside (Ech, blue bar), human TRPM8 (hTRPM8) in response to 500 μM menthol (red bar) or co-application of 50 μM echinacoside (Ech, blue bar). Right panel, current–voltage curves of hTRPs in response to voltage ramps from −100 to +100 mV from the left panel under control conditions (1) after addition of agonists (2) and co-addition of 50 μM echinacoside (3) and wash out or inhibited by 20 μM RR or 130 mM BaCl_2_ (4). (**F**) Summary of average current inhibition of hTRPV3 (red), hTRPA1 (brown), hTRPV1 (purple), hTRPV4 (pink), and hTRPM8 (blue) channels by 50 µM echinacoside (*n* = 5, **** *p* < 0.0001, by unpaired *t* test). Data are expressed as the mean ± SD.

To determine the selectivity of echinacoside, we tested its effects on several other thermo-TRP channels, including TRPA1, TRPV1, TRPV4, and TRPM8. Echinacoside at 50 µM inhibited the human TRPV3 current induced by 2-APB at 50 µM about 79.36 ± 5.9% (Figure 4A,F). In contrast, the same concentration of echinacoside exhibited no inhibition for human TRPA1 (−3.4 ± 4.4%, *p* < 0.0001) or a slight inhibition for human TRPV1 (11.8 ± 1.4%, *p* < 0.0001), human TRPV4 (1.7 ± 1.1%, *p* < 0.0001), and human TRPM8 (11.4 ± 12.9%, *p* < 0.0001) (Figure 4B–F). These results indicate that echinacoside is a relatively selective inhibitor of the TRPV3 channel over other tested thermo-TRP channel subtypes, such as TRPA1, TRPV1, TRPV4, and TRPM8 channels.

### 2.4. Identification of Residues Critical for Echinacoside Binding to TRPV3

**Figure 5:**
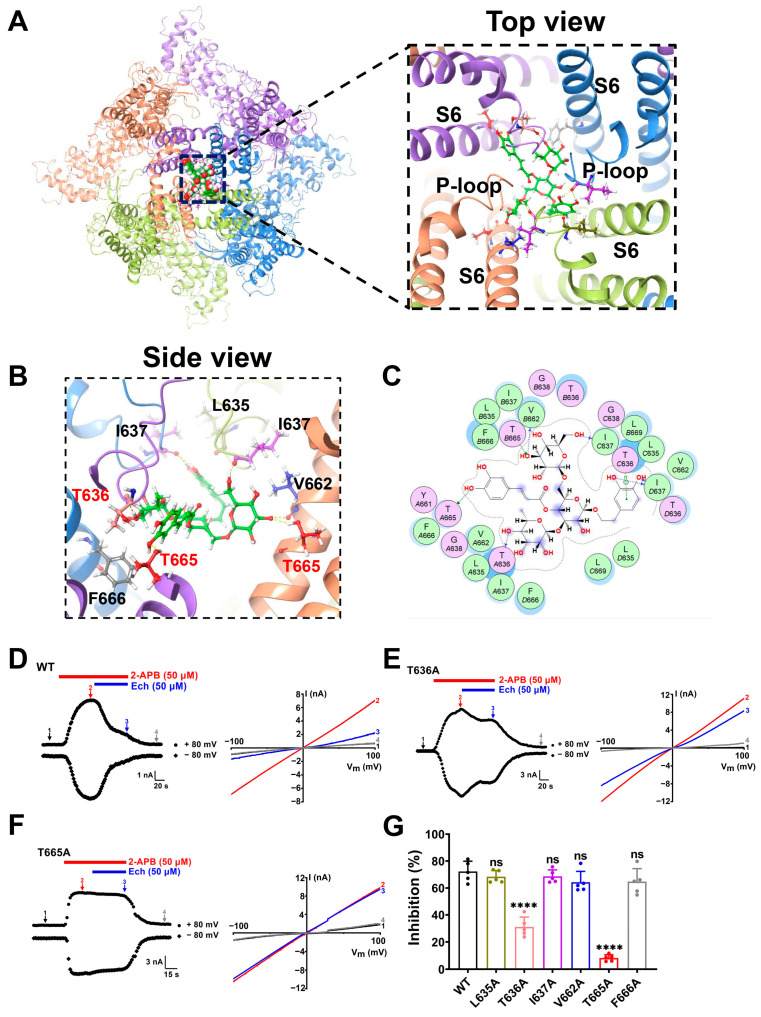
Identification of the key residues for echinacoside binding to the TRPV3 channel. (**A**) Left panel, top view of a putative pocket for echinacoside binding to the mouse TRPV3 structure (PDB ID: 6DVY) from docking. Right panel, the magnified area of the top view of the echinacoside binding pocket. The four subunits of the tetramer are distinguished in four different colors, with bound echinacoside shown in green. (**B**) The magnified area of the side view of the binding site of echinacoside with TRPV3, with residues near the binding pocket colored as follows: L635 (brown), T636 (pink), I637 (purple), V662 (blue), T665 (red), and F666 (gray). Hydrogen bonds are shown as yellow dotted lines. (**C**) Schematic diagram of the 2D interaction between echinacoside and TRPV3. (**D**–**F**) Left panel, whole-cell currents of wild-type (WT) TRPV3 (**D**), T636A (**E**), or T665A (**F**) mutants expressed in HEK293T cells in responses to 2-APB (50 μM, red bar) alone and co-application of 50 μM echinacoside (Ech, blue bar). Right panel, current–voltage curves of WT TRPV3 and mutations in response to voltage ramps from −100 to +100 mV from the left panel under control condition (1) after addition of 2-APB (2) and co-addition of 50 µM echinacoside (3) and washout (4). (**G**) Summary of WT TRPV3 (black) and mutants (L635A, brown; T636A, pink; I637A, purple; V662A, blue; T665A, red; F666A, gray) channel currents inhibited by 50 µM echinacoside (*n* = 5, ns, no significance, **** *p* < 0.0001, by unpaired *t* test). Data are expressed as the mean ± SD.

To identify the residues critical for TRPV3 inhibition by echinacoside, we utilized the cryo-electron microscopy (cryo-EM) structure of mouse TRPV3 bound to 2-APB (Protein Data Bank (PDB) ID: 6DVY) and performed the docking of echinacoside into the structure using Schrödinger software (Maestro 2015). The docking results indicate that echinacoside binds in the central cavity near the pore helix and S6 segment with a docking score of −8.86 (Figure 5A, left panel). The magnified area of the top view reveals that the molecular orientation of echinacoside is well-suited for the TRPV3 ion channel entrance (Figure 5A, right panel). The sugar moiety at the center of the echinacoside molecule acts as a “trunk” supporting the molecular structure, while the caffeoyl group, phenyl ethanol moiety, and two side-chain sugar units function like four “tentacles” firmly grasping TRPV3. The magnified area of the side view (Figure 5B) and the 2D interaction diagram (Figure 5C) further illustrate this interaction clearly. One hydroxyl group of the caffeoyl moiety of echinacoside forms a hydrogen bond with residue T665. The hydroxyl groups on the two side-chain sugar moiety form hydrogen bonds with four residues T636, I637, V662, and T665, respectively. In the phenyl ethanol moiety, besides the two hydroxyl groups on the phenyl ring that interact with residue I637 through hydrogen bonds, the phenyl ring also exhibits a π-H weak interaction with residue T636.

To further confirm the importance of these residues interacting with echinacoside, we generated six alanine mutations: L635A, T636A, I637A, V662A, T665A, and F666A. As shown in Figure 5D–G and Figure S1, mutating two residues T636A and T665A individually markedly reduced echinacoside-induced inhibition of macroscopic TRPV3 currents to 31.1 ± 7.3% (*p* < 0.0001) and 8.2 ± 2.4% (*p* < 0.0001), respectively, as comparted with 72.2 ± 7.6% inhibition for the wild-type (WT) TRPV3 currents. These results indicate that the residues T636 in the p-loop and T665 in the S6 segment of the TRPV3 channel are critical for interaction with echinacoside.

## 3. Discussion

In this study, we confirmed that the natural phenylethanoid glycosides echinacoside derived from traditional Chinese medicine Cistanche Herba effectively alleviates UVB-induced acute skin injury. We further demonstrated that echinacoside is a novel and selective inhibitor of the TRPV3 channel. In addition, molecular docking and site-specific mutagenesis elucidated the pocket formed by the pore loop and the S6 segment and the crucial residues involved in the interaction between echinacoside and TRPV3.

In our recent study, we demonstrated that the TRPV3 channel plays a significant role in UVB-induced skin lesions [28]. Besides the TRPV3 channel, other thermo-TRP channels, such as TRPA1, TRPV1, and TRPV4, have also been reported for their involvement in UVB-induced acute skin injury and sunburn pain [31,32,33]. In this study, evaluation of mouse skin condition showed that TRPV3-deficient mice display a significant attenuation of skin lesions, epidermal thickening, and ear swelling induced by UVB radiation, and echinacoside had no additional protective effect in the *Trpv3* KO mice (Figure 2). We further identified echinacoside as a novel selective inhibitor of the TRPV3 channel over other thermo-TRP channel subtypes, such as the TRPA1, TRPV1, TRPV4, and TRPM8 channels (Figure 4). These findings suggest that echinacoside mitigates UVB-induced acute skin injury by directly targeting and selectively inhibiting TRPV3 channels.

Currently, the binding sites of natural TRPV3 inhibitors, such as scutellarein, isochlorogenic acid A (IAA), and isochlorogenic acid B (IAB), are mainly located near the selectivity filter, and the residue F666 located at the S6 segment, which is important for the channel opening, interacts with these inhibitors [34,35]. Our molecular docking indicated that echinacoside has a strong binding affinity for TRPV3 in the central cavity pocket near the pore helix and S6 segment. The upper portion of the selectivity filter, which faces the extracellular membrane, has a larger pore space, allowing echinacoside to enter and bind to T636 and T665, leading to a conformational change in TRPV3 and closing the channel gate (Figure 5A–C). Subsequent site-specific mutagenesis also demonstrated that two residues T636 and T665 located at near the selectivity filter are crucial for the binding of echinacoside to TRPV3 (Figure 5D–G). Interestingly, the inhibition effect of echinacoside on mutant F666A is similar to that on the WT TRPV3 (Figure 5G, Figure S1). This subtle difference between the critical residues at the binding site may be due to differences in molecular spatial conformation and side-chain groups. Despite minor differences, echinacoside, IAA, and IAB might all bind at the selectivity filter position, triggering the S6 helix to undergo a transition to a closed conformation (α-helix) from an open state (π-helix), thus preventing calcium ion influx.

## 4. Materials and Methods

### 4.1. Animals

Seven-week-old male C57BL/6J mice were purchased from Beijing Vital River Laboratory Animal Technology Co., Ltd. (Beijing, China). Systematic *Trpv3*-knockout (KO) mice were purchased from Cyagen Biosciences Inc. (Suzhou, China) and bred at the Animal Center of Qingdao University. *Trpv3* KO mice were genotyped using polymerase chain reaction according to the protocol provided by Cyagen Biosciences Inc. (Tables S1–S3, Figure S2). Animals were housed in standard laboratory conditions with 12/12 h light–dark and sufficient food and water. All in vivo experiments were carried out at least 7 days after their acclimation to the new environment. Mice were anesthetized with isoflurane (1–1.5%) using a gas anesthesia device and were sacrificed under anesthesia. All animal procedures were approved by the Institutional Animal Care and Use Committee of Qingdao University Medical College (protocol code QDU-AEC-2024158), approval date 1 March 2024.

### 4.2. Compounds and Creams

The natural compound echinacoside (MW: 786.73) was purchased from Desite Biological Technology Co., Ltd. (Chengdu, China). Vitamin E was purchased from Shanghai Yuanye Bio-Technology Co., Ltd. (Shanghai, China). Compounds 2-aminoethoxydiphenylborate (2-APB), capsaicin, menthol, barium chloride (BaCl_2_), GSK1016790A (GSK101), and allyl isothiocyanate (AITC) were purchased from Sigma Aldrich (St. Louis, MO, USA). The purity of each standard compound was no less than 98% by high-performance liquid chromatography analysis. All compounds, except for vitamin E, were prepared as stock solutions dissolved in dimethyl sulfoxide (DMSO). For patch-clamp recordings, compounds were diluted in normal perfusion solution. This study was performed using a w/o cream formulation containing 1,2-propanediol as the main water phase, and octadecanoic acid, 1-hydroxyoctadecane, liquid paraffin, and glycerol monostearate as the main oil phase. For the preparation of echinacoside cream, echinacoside was first dissolved in 1,2-propanediol, then this solution was added to the other water phase components. In preparing vitamin E cream, vitamin E was dissolved in the oil phase components. Subsequently, the aqueous phase was added to the oil phase at 80 °C, stirred to achieve complete emulsification, and cooled to obtain two creams. The drug loading capacities of echinacoside and vitamin E in the creams were 0.2%, 1%, 5%, and 5%, respectively.

### 4.3. Establishment and Treatment of UVB-Induced Acute Skin Injury Mouse Model

Prior to establishing the UVB-induced acute skin injury mouse model, mice were shaved in advance and were given one day to acclimate environment. The establishment of the UVB-induced acute skin injury mouse model was conducted based on previous studies [28]. Mice were treated with different concentrations of echinacoside or vitamin E immediately after a single exposure to 500 mJ/cm^2^ UVB irradiation. Each mouse in the treatment groups received an even application of 0.1 g of the corresponding cream on the depilated area of the back (2 cm × 3 cm), and an application of 0.02 g of the corresponding cream was administered to the exposed areas on both sides of each ear (1 cm × 0.5 cm). For the next four days, the corresponding cream was applied locally to the mice in each treatment group.

### 4.4. Skin Lesion Scoring and Dorsal Skin Thickness Measurements

In the acute skin injury mouse model, dorsal skin thickness was measured immediately and every 24 h after UVB radiation for five consecutive days using a vernier caliper. For comprehensive dermatitis scoring, dermatitis severity was assessed according to the following four symptoms: (1) erythema/hemorrhage; (2) scarring/dryness; (3) edema; and (4) excoriation/erosion. Each symptom was scored from 0 to 3 (0, none; 1, mild; 2, moderate; and 3, severe). Total score was defined as the sum of the individual scores, ranging from 0 to 12.

### 4.5. Histology

Mouse dorsal skin tissues were fixed in 4% paraformaldehyde overnight before being embedded in paraffin. Mouse ear skin tissues were fixed in 4% paraformaldehyde before dehydrated in 30% sucrose for 48 h and embedded with optimal cutting temperature compound (Sakura, Torrance, CA, USA) and sectioned using a freezing microtome (Leica, Nussloch, Germany). Tissue samples were stained with hematoxylin–eosin staining (H&E). Imaging of tissue sections was performed using a panoramic tissue cell scanning analyzer (Pannoramic MIDI, Budapest, Hungary).

### 4.6. Cell Culture and Transient Transfection

Human embryonic kidney (HEK) 293T cells were cultured in Dulbecco’s modified Eagle medium (DMEM, Gibco, ThermoFisher, Grand Island, NY, USA) supplemented with 10% fetal bovine serum (FBS, PAN-Biotech, Aidenbach, Germany) at 37 °C and 5% CO_2_. HEK293T cells were digested with trypsin and inoculated onto glass slides for 24 h before transfection with the plasmid vectors containing hTRPV3 (NM_145068.4), hTRPA1 (NM_007332.3), hTRPV1 (NM_080704.3), hTRPV4 (NM_021625.5), and hTRPM8 (NM_024080.5) cDNA using Lipofectamine 2000 (Invitrogen, Carlsbad, CA, USA). Patch clamp recording of HEK293T cells was performed 18 h after transfection.

### 4.7. Electrophysiological Recordings

Patch clamp recording was completed using an EPC 10 USB amplifier and Patchmaster software (HEKA Harvard, Holliston, Church Hill, TN, USA). The borosilicate glass pipettes were fabricated using a vertical micropipette puller (PC-100, Narishige, Tokyo, Japan) to achieve tip resistances of 3–5 MΩ for whole-cell or 6–10 MΩ for inside-out patch clamp recordings when filled with the pipette solution. Pipette solution and bath solution both contained (in mM): 130 NaCl, 3 HEPES, and 0.2 EDTA (pH = 7.4). Whole-cell patch clamp recording showed that the membrane potential was maintained at 0 mV, currents were recorded at a slope voltage ramp from −100 to +100 mV for 500 ms and analyzed at ± 80 mV. For inside-out patch recordings, membrane potential was clamped at a voltage of −60 mV to observe the opening of the single channel. The current sampling frequency was 20 kHz, filtered at 2.0 kHz.

### 4.8. Molecular Docking and Site-Directed Mutagenesis

Molecular docking was performed using Schrödinger Glide (Maestro software suite 2015, Schrödinger, New York, NY, USA). The two-dimensional (2D) and three-dimensional (3D) structures of echinacoside were generated using ChemBioDraw Ultra 14.0 (Cambridge Soft) and ChemBio3D Ultra 14.0 (Cambridge Soft), respectively. The cryo-electron microscopy structure of the mTRPV3 protein bound to 2-APB (PDB ID: 6DVY) was obtained from the Protein Data Bank. Molecular docking between echinacoside and TRPV3 was performed using Schrödinger Glide with default parameters. Prior to semi-flexible docking, both the compound and protein underwent energy minimization and optimization through the built-in LigPrep module. The docking of echinacoside with TRPV3 was performed based on the reported binding sites of TRPV3 inhibitors, and the results were ranked according to their docking scores. Based on its highest score, the pocket located between the pore helix and S6 segment of TRPV3 was selected as the binding site for echinacoside. All TRPV3 mutations were synthesized using the Mut Express II Fast Mutagenesis Kit V2 (Vazyme, Nanjing, China) according to the manufacturer’s instructions. All mutants were sequenced to confirm the correct synthesis of the mutation.

### 4.9. Statistical Analysis

All data are expressed as the mean ± standard deviation (SD). Statistical significance was determined using the Shapiro–Wilk normality test before applying the unpaired *t* test; one-way and two-way ANOVA followed by a multiple-comparison test was used to evaluate statistical significance using GraphPad Prism 7.0 software (La Jolla, CA, USA). A value of *p* < 0.05 was considered statistically significant.

## 5. Conclusions

In conclusion, our data demonstrate that echinacoside, the main active component of Cistanche Herba, is a selective inhibitor of TRPV3. Echinacoside directly inhibits TRPV3 by binding to the central cavity pocket near the pore helix and S6 segment of TRPV3, thereby ameliorating UVB-induced skin damage. Our data further confirm that TRPV3 represents a potential therapeutic target for UVB radiation-induced skin damage. Our findings not only unveil a novel role of echinacoside isolated from traditional Chinese medicine Cistanche Herba in inhibiting TRPV3, but also provide a new tool and guidance for further research into the mechanism of echinacoside in treating irradiation-related skin diseases and exploring the downstream signaling pathway. 

## Data Availability

Data are contained within the manuscript.

## Supplementary Materials

The following supporting information can be downloaded at: https://www.mdpi.com/article/10.3390/molecules30092026/s1, Figure S1: The inhibitory effect of echinacoside on TRPV3 mutations. Table S1: The primer sequences used in the PCR. Table S2: The components of the PCR reaction system. Table S3: The conditions for the PCR reaction. Figure S2: Representative image of agarose gel electrophoresis for identification of *Trpv3* knockout mice.

## Abbreviations

2-APB, 2-aminoethoxydiphenyl borate; 2D, two-dimensional; 3D, three-dimensional; AITC, allyl isothiocyanate; Cryo-EM, cryo-electron microscopy; DMEM, Dulbecco’s modified Eagle’s medium; DMSO, dimethyl sulfoxide; Ech, echinacoside; FBS, fetal bovine serum; GSK101, GSK1016790A; H&E, hematoxylin and eosin; HEK293T, human embryonic kidney 293T; IAA, isochlorogenic acid A; IAB, isochlorogenic acid B; KO, knockout; PDB, protein data bank; SD, standard deviation; TRP, transient receptor potential; TRPA1, TRP ankyrin 1; TRPM8, TRP melastatin 8; TRPV1, TRP vanilloid 1; TRPV4, TRP vanilloid 4; UVA, ultraviolet A; UVB, ultraviolet B; UVR, ultraviolet radiation; VE, vitamin E; WT, wild-type.

## References

[1] Hwa C., Bauer E.A., Cohen D.E. (2011). Skin biology. Dermatol. Ther..

[2] Jiao Q., Zhi L., You B., Wang G., Wu N., Jia Y. (2024). Skin homeostasis: Mechanism and influencing factors. J. Cosmet. Dermatol..

[3] Passeron T., Lim H.W., Goh C.L., Kang H.Y., Ly F., Morita A., Ocampo Candiani J., Puig S., Schalka S., Wei L. (2021). Photoprotection according to skin phototype and dermatoses: Practical recommendations from an expert panel. J. Eur. Acad. Dermatol. Venereol..

[4] Trakatelli M., Barkitzi K., Apap C., Majewski S., De Vries E. (2016). Skin cancer risk in outdoor workers: A European multicenter case–control study. J. Eur. Acad. Dermatol. Venereol..

[5] Kammeyer A., Luiten R.M. (2015). Oxidation events and skin aging. Ageing Res. Rev..

[6] Matsumura Y., Ananthaswamy H.N. (2004). Toxic effects of ultraviolet radiation on the skin. Toxicol. Appl. Pharmacol..

[7] Sarkar S., Gaddameedhi S. (2018). UV-B-Induced Erythema in Human Skin: The Circadian Clock Is Ticking. J. Investig. Dermatol..

[8] Khalil C. (2018). Human skin explants an in vitro approach for assessing UVB induced damage. Toxicol. In Vitro.

[9] Cavinato M., Waltenberger B., Baraldo G., Grade C.V.C., Stuppner H., Jansen-Dürr P. (2017). Plant extracts and natural compounds used against UVB-induced photoaging. Biogerontology.

[10] Lei D., Ye L., Wen S., Zhang J., Zhang L., Man M.-Q. (2024). Preventive and Therapeutic Benefits of Natural Ingredients in Photo-Induced Epidermal Dysfunction. Skin Pharmacol. Physiol..

[11] Milutinov J., Pavlović N., Ćirin D., Atanacković Krstonošić M., Krstonošić V. (2024). The Potential of Natural Compounds in UV Protection Products. Molecules.

[12] Song Y., Zeng K., Jiang Y., Tu P. (2021). Cistanches Herba, from an endangered species to a big brand of Chinese medicine. Med. Res. Rev..

[13] Li Z., Lin H., Gu L., Gao J., Tzeng C.-M. (2016). Herba Cistanche (Rou Cong-Rong): One of the Best Pharmaceutical Gifts of Traditional Chinese Medicine. Front. Pharmacol..

[14] Jin X., Qiao L., Mei S., Zhang H., Ji S., Wang N. (2017). Herba Cistanches: Anti-aging. Aging Dis..

[15] Zhou S., Feng D., Zhou Y., Duan H., Jiang Y., Yan W. (2023). Analysis of the active ingredients and health applications of cistanche. Front. Nutr..

[16] Trampetti F., Pereira C., Rodrigues M.J., Celaj O., D’Abrosca B., Zengin G., Mollica A., Stefanucci A., Custódio L. (2019). Exploring the halophyte *Cistanche phelypaea* (L.) Cout as a source of health promoting products: In vitro antioxidant and enzyme inhibitory properties, metabolomic profile and computational studies. J. Pharm. Biomed. Anal..

[17] Zou P., Song Y., Lei W., Li J., Tu P., Jiang Y. (2017). Application of 1 H NMR-based metabolomics for discrimination of different parts and development of a new processing workflow for *Cistanche deserticola*. Acta Pharm. Sin. B.

[18] Zhang D., Lu C., Yu Z., Wang X., Yan L., Zhang J., Li H., Wang J., Wen A., Singhal S.S. (2017). Echinacoside Alleviates UVB Irradiation-Mediated Skin Damage via Inhibition of Oxidative Stress, DNA Damage, and Apoptosis. Oxid. Med. Cell. Longev..

[19] Wen S.Y., Ng S.C., Noriega L., Chen T.J., Chen C.J., Lee S.D., Huang C.Y., Kuo W.W. (2025). Echinacoside promotes collagen synthesis and survival via activation of IGF-1 signaling to alleviate UVB-induced dermal fibroblast photoaging. Biofactors.

[20] Kalinovskii A.P., Utkina L.L., Korolkova Y.V., Andreev Y.A. (2023). TRPV3 Ion Channel: From Gene to Pharmacology. Int. J. Mol. Sci..

[21] Lei J., Tominaga M. (2024). Unlocking the therapeutic potential of TRPV3: Insights into thermosensation, channel modulation, and skin homeostasis involving TRPV3. Bioessays.

[22] Su W., Qiao X., Wang W., He S., Liang K., Hong X. (2023). TRPV3: Structure, Diseases and Modulators. Molecules.

[23] Xu H.X., Ramsey I.S., Kotecha S.A., Moran M.M., Chong J.H.A., Lawson D., Ge P., Lilly J., Silos-Santiago I., Xie Y. (2002). TRPV3 is a calcium-permeable temperature-sensitive cation channel. Nature.

[24] Lai-Cheong J.E., Sethuraman G., Ramam M., Stone K., Simpson M.A., McGrath J.A. (2012). Recurrent heterozygous missense mutation, p.Gly573Ser, in the TRPV3 gene in an Indian boy with sporadic Olmsted syndrome. Br. J. Dermatol..

[25] Seo S.H., Kim S., Kim S.-E., Chung S., Lee S.E. (2020). Enhanced Thermal Sensitivity of TRPV3 in Keratinocytes Underlies Heat-Induced Pruritogen Release and Pruritus in Atopic Dermatitis. J. Investig. Dermatol..

[26] Um J.Y., Kim H.B., Kim J.C., Park J.S., Lee S.Y., Chung B.Y., Park C.W., Kim H.O. (2022). TRPV3 and Itch: The Role of TRPV3 in Chronic Pruritus according to Clinical and Experimental Evidence. Int. J. Mol. Sci..

[27] Nattkemper L.A., Lipman Z.M., Ingrasci G., Maldonado C., Garces J.C., Loayza E., Yosipovitch G. (2023). Neuroimmune Mediators of Pruritus in Hispanic Scalp Psoriatic Itch. Acta Derm. Venereol..

[28] Qu Y., Sun X., Wei N., Wang K. (2023). Inhibition of cutaneous heat-sensitive Ca^2+^-permeable transient receptor potential vanilloid 3 channels alleviates UVB-induced skin lesions in mice. FASEB J..

[29] Zhang H., Sun X., Qi H., Ma Q., Zhou Q., Wang W., Wang K. (2019). Pharmacological Inhibition of the Temperature-Sensitive and Ca^2+^-Permeable Transient Receptor Potential Vanilloid TRPV3 Channel by Natural Forsythoside B Attenuates Pruritus and Cytotoxicity of Keratinocytes. J. Pharmacol. Exp. Ther..

[30] Sun X., Qi H., Wu H., Qu Y., Wang K. (2020). Anti-pruritic and anti-inflammatory effects of natural verbascoside through selective inhibition of temperature-sensitive Ca2+-permeable TRPV3 channel. J. Dermatol. Sci..

[31] Camponogara C., Brum E.S., Pegoraro N.S., Brusco I., Rocha F.G., Brandenburg M.M., Cabrini D.A., André E., Trevisan G., Oliveira S.M. (2020). Neuronal and non-neuronal transient receptor potential ankyrin 1 mediates UVB radiation-induced skin inflammation in mice. Life Sci..

[32] Huang K.F., Ma K.H., Chang Y.J., Lo L.C., Jhap T.Y., Su Y.H., Liu P.S., Chueh S.H. (2019). Baicalein inhibits matrix metalloproteinase 1 expression via activation ofTRPV1-Ca-ERKpathway in ultraviolet B–irradiated human dermal fibroblasts. Exp. Dermatol..

[33] Moore C., Cevikbas F., Pasolli H.A., Chen Y., Kong W., Kempkes C., Parekh P., Lee S.H., Kontchou N.-A., Yeh I. (2013). UVB radiation generates sunburn pain and affects skin by activating epidermal TRPV4 ion channels and triggering endothelin-1 signaling. Proc. Natl. Acad. Sci. USA.

[34] Wang Y., Tan L., Jiao K., Xue C., Tang Q., Jiang S., Ren Y., Chen H., El-Aziz T.M.A., Abdelazeem K.N.M. (2022). Scutellarein attenuates atopic dermatitis by selectively inhibiting transient receptor potential vanilloid 3 channels. Br. J. Pharmacol..

[35] Qi H., Shi Y., Wu H., Niu C., Sun X., Wang K. (2022). Inhibition of temperature-sensitive TRPV3 channel by two natural isochlorogenic acid isomers for alleviation of dermatitis and chronic pruritus. Acta Pharm. Sin. B.

