# Supplementary material for: Echinacoside Ameliorates UVB-Induced Skin Damage Through Selective Inhibition of the Cutaneous TRPV3 Channel
Source: Molecules. 2025 May 2;30(9):2026. doi: 10.3390/molecules30092026 (PMC12073194; doi:10.3390/molecules30092026)
Supplement: Supplementary file 1 [file molecules-30-02026-s001.zip › molecules-3556382-supplementary.pdf]

Supplementary information

Echinacoside Ameliorates UVB-Induced Skin Damage Through Selective Inhibition of the Cutaneous TRPV3 Channel

Shilun Mo[1,†] Xinying Yue[1,†], Yaxuan Qu[2], Guoji Zhang[2], Liqin Wang[1], Xiaoying Sun[1,3,*]

[1]Department of Natural Medicinal Chemistry and Pharmacognosy, School of Pharmacy, Qingdao Medical College of Qingdao University, 1 Ningde Road, Qingdao 266073, China;
[2]Department of Pharmacology, School of Pharmacy, Qingdao Medical College of Qingdao University, 1 Ningde Road, Qingdao 266073, China;
[3]Institute of Innovative Drugs, Qingdao University, 38 Dengzhou Road, Qingdao 266021, China

[*] Correspondence: xiaoyingsun@qdu.edu.cn
[†] These authors contributed equally to this work.

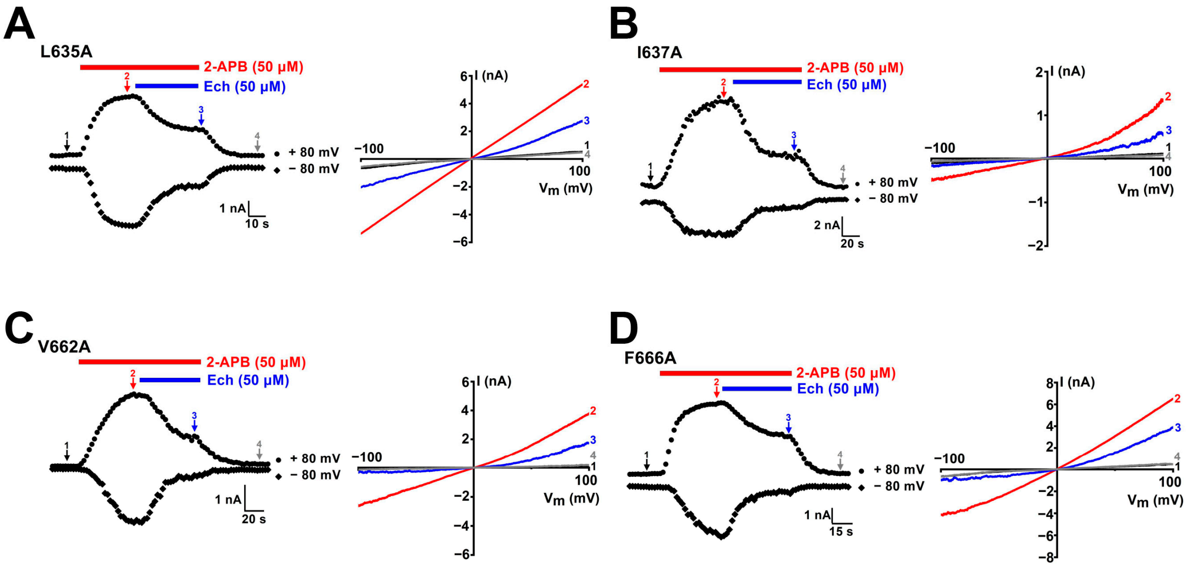

Figure S1. The inhibitory effect of echinacoside on TRPV3 mutations. (A-D) Left panel, representative whole-cell recordings of L635A (A), I637A (B), V662A (C) and F666A (D) mutants expressed in HEK293T cells in the presence of 50 μM 2-APB alone (red bar) and co-application of 50 μM echinacoside (Ech, blue bar). Right panel, current-voltage curves of mutations in response to voltage ramps from −100 to +100 mV under control condition (1) after addition of 50 μM 2-APB (2) and co-addition of 50 μM echinacoside (3) and washout (4).

Table S1. The primer sequences used in the PCR.

**Primers: (Annealing Temperature 60.0 ºC)**	**Forward 5'-3'**	**Reverse 5'-3**
Primer 1	CACATAATGCACCCTGGTCACTCT	GAAGCTGTCCTCAAAGATTCCCAGAC
Primer 2	CACATAATGCACCCTGGTCACTCT	ACAAGCAACATCAAGTGGTGGTAAG

Table S2. The components of the PCR reaction system.

**Component**	**×1**
ddH_2O	9.0 μl
Product primer F	1.0 μl
Product primer R	1.0 μl
Premix Taq	12.5 μl
DNA	1.5 μl
Total	25 μl

Table S3. The conditions for the PCR reaction.

**Step**	**Temp.**	**Time**	**Cycles**
Initial denaturation	94 ℃	3 min	
Denaturation	94 ℃	30 s	35×
Annealing	60 ℃	30 s	
Extension	72 ℃	30 s	
Additional extension	72 ℃	5 min	

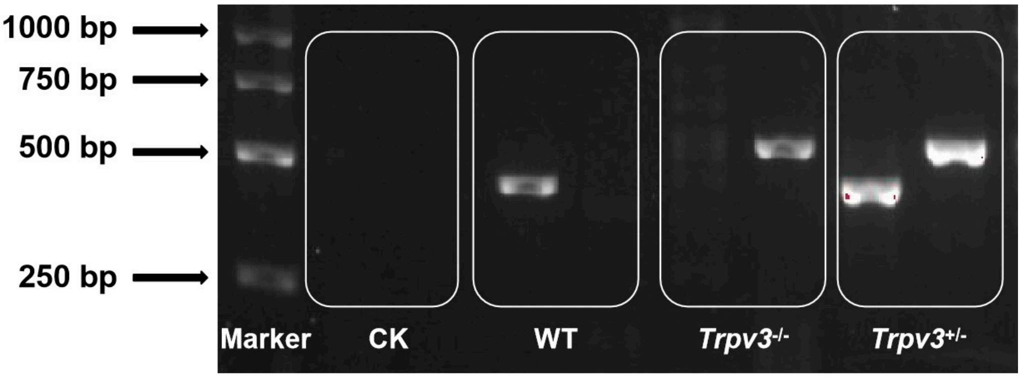

Figure S2. Representative image of agarose gel electrophoresis for identification of *Trpv3* knockout mice. The solvent control group (CK) shows no band. The wild-type (WT) mice show a single band at 434 bp. The *Trpv3* knockout (*Trpv3*$^{-/-}$) mice show a single band at 544 bp. The heterozygous (*Trpv3*$^{+/-}$) mice show two bands at 434 bp and 544 bp, respectively.
